# An easy way to improve lab meetings

**DOI:** 10.7554/eLife.111095

**Published:** 2026-03-18

**Authors:** Cara Glynn, Tiago Monteiro

**Affiliations:** 1 School of Life Sciences, University of Lincoln Lincoln United Kingdom; 2 Domestication Lab, Konrad Lorenz Institute of Ethology, Department of Interdisciplinary Life Sciences, University of Veterinary Medicine Vienna Vienna Austria

**Keywords:** research culture, lab meetings, early-career researchers, None

## Abstract

Sharing positive and negative experiences at lab meetings can make a career in science a little less hard, a little more pleasant, and a little more human.

Most scientists are part of a research lab, and irrespective of career stage, lab meetings are a good way to get acquainted with colleagues and to learn about how their research is going. However, lab meetings can also be intimidating, particularly for early-career researchers, and this feeling tends to be proportional to the size of the group and to the seniority of those attending. Moreover, some people can find it challenging to contribute meaningfully to lab meetings as they are often dominated by more senior members of the group or by people with more extroverted personalities.

We are both associated with the Domestication Lab at the University of Veterinary Medicine in Vienna. There are about 20 people in the group – including two senior scientists, three postdocs, nine PhD students, and a fluctuating number of master’s students and interns – and the lab meets as a group every week during term time. Research in the lab is a mix of field work (with free-ranging dogs in Morocco, and wild wolves in both Italy and the US), and lab-based work (with pet dogs and other captive canids). One of us [TM] is a postdoc, who leads a subgroup of around six people whose work focuses on touchscreen-based experiments looking into behavioural and cognitive processes. This subgroup also meets every week.

Over the past year or so, we have tried to make the meetings of this subgroup more inclusive – and more of an opportunity for team building – by asking everyone at the meeting to share one positive event and one negative event that has happened since the previous meeting. Participation was encouraged but voluntary, and the events could be work-related, personal, or both. This exercise – which we light-heartedly named *Ups & Downs* – always happens at the start of the meeting.

The most striking outcome of this was that everyone (including the more introverted members of the group) was willing to share something. Moreover, even when time was limited, the team would oppose skipping this part of the meeting, as it had quickly become a highlight. Initially, *Ups & Downs* was intended solely as a unifying activity for the group, and the records kept were for reflective purposes. However, as time went on, we realised that our experience could be valuable to share with the community.

Across 41 meetings, just over half (54%) of the positive events were professional, with 24% being personal, and 22% being both. For negative events, 40% were professional, 36% were personal, and 24% were both. Common themes shared in the personal sphere were the enjoyment of holidays and the burden of tiredness. Examples in the professional realm included the satisfaction of submitting a paper to a journal, and its flip side – having a paper rejected by a journal. Topics that bridged both the personal and the professional included the excitement of starting a new role or course, and the challenges of maintaining a healthy work-life balance.

We believe that the greatest benefit of *Ups & Downs* was that it encouraged each team member to actively think about something positive that happened recently. At the same time, the fact that everyone also shared a negative event created a sense of openness and camaraderie, helping others realise that everyone struggles at times. These moments fostered empathy within the group, enabling colleagues to offer personal insights and suggest solutions to each other’s difficulties.

Anecdotally, as time went on, we also noticed an increase in the frequency of more personal issues being shared, highlighting the important role that building trust plays in such teams. Further to this, some team members mentioned that the activity enabled them to develop their confidence to speak in group settings, which has since benefitted them in both personal and professional life.

Admittedly, *Ups & Downs* has added a quite a lot of time to our meetings, and the bigger the group, the longer this time becomes. Nevertheless, we feel that this is time well spent, because we are a stronger and more connected team now than we ever were before. A testament to this is the fact that former group members continue to take the time to attend these meetings whenever they can (in person or remotely). For larger groups, it may be more practical to periodically reserve one meeting for an activity like *Ups & Downs* or, alternatively, to carry it out within sub-group meetings, as we do.

A career in science is hard; people’s lives can be unstable, especially early in their careers, and rejection is a constant companion. Making the effort to routinely find something positive to share, while also recognising that everyone struggles at times, helps us feel more optimistic and less isolated. Whether facing the disappointment of a manuscript being once again rejected, or the frustration of a disagreement with a supervisor, knowing that other people care, have experienced similar situations, and above all, have time to listen, makes it a little less hard, a little more pleasant, and a little more human. What this experience has taught us is that sharing is caring, people are worth it, and these meetings were definitely not a waste of anyone’s time!

